# Characteristics of patients with heart failure and normal ejection fraction

**DOI:** 10.20407/fmj.2025-005

**Published:** 2025-08-06

**Authors:** Yusuke Funato, Hideki Kawai, Yuji Kono, Kazuhiro Terashima, Tomoya Ishiguro, Yohei Otaka, Masanobu Yanase, Hideo Izawa

**Affiliations:** 1 Department of Cardiology, Fujita Health University, School of Medicine, Toyoake, Aichi, Japan; 2 Rehabilitation Complex, Fujita Health University Hospital, Toyoake, Aichi, Japan; 3 Department of Rehabilitation, Fujita Health University, School of Medicine, Toyoake, Aichi, Japan

**Keywords:** Heart failure, Sarcopenia, Skeletal muscle index, Ejection fraction

## Abstract

**Objectives:**

A new classification of heart failure based on the effects of medication has recently come into use. According to this classification, heart failure is divided into heart failure with normal ejection fraction (HFnEF; defined as an EF ≥55% for men and ≥60% for women) and non-HFnEF. However, the characteristics of patients with HFnEF are still unclear. Accordingly, in this study, we sought to identify the background characteristics, including non-cardiac factors, of patients with HFnEF.

**Methods:**

We retrospectively divided 304 eligible patients who were hospitalized for worsening heart failure at our institution between December 2020 and December 2022 into an HFnEF group (n=37) and a non-HFnEF group (n=267) and compared their demographic and clinical characteristics.

**Results:**

There were more elderly patients in the non-HFnEF group, along with fewer patients with coronary artery disease and low serum hemoglobin and NT-proBNP levels and a higher proportion of patients with a low skeletal muscle index (<7.0 kg/m^2^ for men and <5.7 kg/m^2^ for women). Multivariate analysis with addition of patient sex identified a low skeletal muscle index (odds ratio 2.96, p<0.01) to be an independent determinant of HFnEF along with older age and low NT-proBNP.

**Conclusions:**

A low skeletal muscle index was significantly more common in patients with HFnEF than in those with non-HFnEF. Intensive nutrition and exercise therapy to increase skeletal muscle mass may improve the prognosis in patients with HFnEF who respond poorly to standard pharmacological treatment.

## Introduction

Previous studies have shown that the effects of medication for heart failure depend on the left ventricular ejection fraction (LVEF), with the prognostic impact of medical therapy becoming weaker as LVEF increases.^1^ Patients with an LVEF >50% are typically defined as having HF with preserved EF. However, a recent review found that men with an LVEF of ≥55% and women with a LVEF of ≥60% are often resistant to medication for heart failure. These patients have now been categorized as having heart failure with normal ejection fraction (HFnEF).^2^

HFnEF has been considered to have a heterogeneous pathology and to be a condition in which various etiologies are combined in various proportions.^3^ The etiology of HFnEF could include factors other than heart disease, such as chronic sympathetic nervous activation, upregulated inflammatory pathways, and sarcopenia. However, the characteristics of patients with HFnEF remain unclear. Accordingly, in this study, we aimed to determine the characteristics of patients with HFnEF by comparing background features between patients with HFnEF and those with non-HFnEF, including non-cardiac factors such as sarcopenia.

## Methods

### Study population

**Figure 1:**
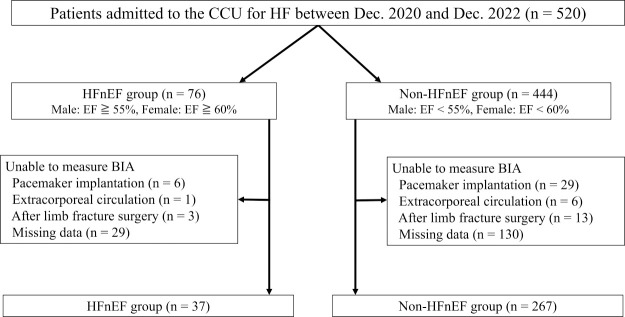
Flow diagram showing the patient selection procedure. BIA, bioelectrical impedance analysis; CCU, coronary care unit; EF, ejection fraction; HF, heart failure; HFnEF, heart failure with normal ejection fraction

We retrospectively searched the medical records at our hospital and identified 520 consecutive patients who were admitted with worsening heart failure between December 2020 and December 2022. After exclusion of 216 patients who could not undergo bioelectrical impedance measurements to calculate the skeletal muscle index (SMI) because of pacemaker implantation, extracorporeal circulation, surgery for limb fracture, or missing data (Figure 1), the remaining 304 patients were divided into an HFnEF group and a non-HFnEF group. The HFnEF group included men with an LVEF of ≥55% and women with an LVEF of ≥60% on echocardiography, while the non-HFnEF group included men with an LVEF of <55% and women with an LVEF of <60%.^2^ An echocardiogram was obtained from all patients on admission to hospital. Chronic kidney disease was defined as a glomerular filtration rate of <60 mL/min/1.73 m^2^.^4^

The study protocol was approved by the institutional review board of Fujita Health University (identifier HM23-236) and performed in accordance with the Declaration of Helsinki. The requirement for written informed consent was waived in view of the retrospective observational nature of the research.

### Bioelectrical impedance analysis

Bioelectrical impedance analysis (BIA) was performed using the InBody S-10 system (Biospace Co., Ltd., Seoul, Korea). This device is capable of obtaining 15 impedance measurements per patient by measuring the conductance of current across five body segments (legs, arms, and torso) at three frequencies (5, 50, and 500 kHz). All patients underwent BIA in the lying position on the day of admission. We measured the SMI, along with fat, mineral, and protein content, and defined a low SMI as a score of <7.0 kg/m^2^ in men and <5.7 kg/m^2^ in women.^5^ We also calculated the edema index (extracellular water/total body water), which can be used as a reference for evaluating fluid status in patients with heart failure.^6^

### Statistical analysis

Continuous data were tested for normality using the Shapiro–Wilk test and are shown as the mean±standard deviation if normally distributed and as the median [interquartile range] if non-normally distributed. Categorical variables are presented as the frequency (percentage). Differences between the two groups were evaluated using the Mann–Whitney test or Student’s *t*-test for continuous variables and the chi-squared test for categorical variables. Logistic regression analyses were subsequently performed to identify factors that were correlated with HFnEF. During univariate logistic regression analysis, normal amino-terminal pro-brain natriuretic peptide (NT-proBNP), valvular heart disease, coronary artery disease, prescription of diuretics, older age, and a low SMI score were associated with the presence of HFnEF. Multivariate regression analysis was performed after adjusting for NT-proBNP, valvular heart disease, coronary artery disease, prescription of diuretics, older age, low SMI, and patient sex. All statistical analyses were performed using JMP version 13 (SAS Institute Inc., Cary, NC, USA) and p<0.05 was considered significant.

## Results

**Figure 2:**
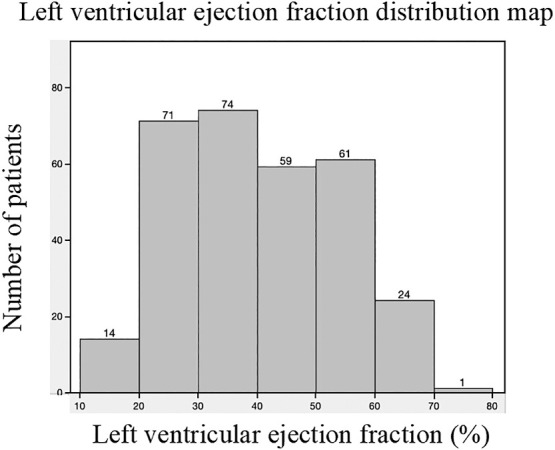
Distribution of left ventricular ejection fraction in the study population on admission.

The characteristics of patients in the HFnEF and non-HFnEF groups are compared in Table 1. There were significant differences in age, prevalence of coronary artery disease, prevalence of valvular heart disease, hemoglobin, NT-proBNP level, prescription of diuretics at admission, and the frequency of low SMI between the two groups. There was no significant between-group difference in New York Heart Association functional class (recorded in the medical notes for only 202 of 304 patients). Given that measurement of SMI by BIA is affected by body fluid volume, we also calculated the edema index and found no significant between-group difference. The distribution of LVEF in the study population on admission is shown in Figure 2.

Univariate analysis identified older age, coronary artery disease, valvular heart disease, NT-proBNP, prescription of diuretics, and low SMI to be significant predictors of HFnEF. Multivariate analysis of these factors, including sex, showed that low SMI, along with age, NT-proBNP, and prescription of diuretics were independent determinants of HFnEF (Table 2).

## Discussion

In this study, patients with HFnEF were older and had less muscle mass than those with non-HFnEF. To our knowledge, this study is the first to identify low skeletal muscle mass by BIA in patients with HFnEF. Lower muscle mass influences sympathetic nerve activity via ergoreceptor activity and neurohumoral factors, causing shortness of breath and exacerbation of heart failure. This mechanism also causes endocrine abnormalities, peripheral circulation failure, inflammation, and oxidative stress, creating a vicious cycle that promotes a decline in skeletal muscle strength.^7^ Older age could be another reason for the lower muscle mass in patients with HFnEF. There is evidence suggesting that lower muscle mass can be caused not only by aging but also factors such as chronic diseases and lifestyle habits. Elderly patients have more comorbidities, which also causes decreased muscle mass in those with HFnEF.^8–10^ Furthermore, lower muscle mass increases the risk of falls and can cause a general decline in physical function. While moderate exercise and a well-balanced diet are recommended for patients with decreased muscle mass, no effective pharmacological treatments are currently available for low muscle mass.^11,12^ Thus, no pharmacological therapy is effective against worsening heart failure attributed to low muscle mass, suggesting the need to consider both nutritional and exercise therapies.^13^ Cardiac rehabilitation for patients with heart failure with preserved ejection fraction has been reported to be effective,^14^ and it is thought that cardiac rehabilitation should also be performed in patients with HFnEF.

This study evaluated skeletal muscle mass using BIA but failed to assess the presence of sarcopenia, which is determined based on grip strength, walking speed, and skeletal muscle mass.^5^ However, grip strength and walking speed may be difficult to measure in the acute stage of worsening heart failure, and problems regarding the accuracy and reproducibility of measurements in elderly patients have been noted as a result of dementia and decreased ability to perform activities of daily living.^15^ BIA is capable of measuring SMI with high reproducibility, even during the acute stage of heart failure. Moreover, studies have shown that increased skeletal muscle mass can improve the prognosis of patients with heart failure.^16^ These findings suggest that performing BIA and providing nutritional and exercise therapies may improve the prognosis in patients with HFnEF.

This study has some limitations. First, it had a retrospective design and included a small number of cases from a single institution, this means that the results of this study may not be generalizable to other populations. Second, BIA was performed for worsening heart failure on the day of admission. Although SMI is considered highly reproducible regardless of heart failure status, the possibility that skeletal muscle mass values were affected by fluid volume cannot be ruled out. Third, only inpatients were investigated, which may have introduced selection bias. Finally, previous studies have reported that older age, hypertension, atrial fibrillation, coronary artery disease, diabetes, and obesity are background factors that are strongly associated with heart failure with preserved ejection fraction. ^17^ Although we included these factors in multivariate analysis, most could not be shown to be significant predictors of HFnEF. However, it is possible that our sample size was too small to detect statistically significant between-group differences in some of these factors.

In conclusion, this study found that the SMI is lower in patients with HFnEF than in those with non-HFnEF. Intensive nutrition and exercise therapies to increase skeletal muscle mass may improve the prognosis of patients with HFnEF who respond poorly to standard pharmacological treatment.

## Figures and Tables

**Table 1:** Patient demographic and clinical characteristics

	HFnEF group (n=37)	Non-HFnEF group (n=267)	p-value
Age (years), median [IQR]	84 [78, 88]	79 [71, 86]	0.0073
Men, n (%)	19 (51)	167 (63)	0.1948
Coronary artery disease, n (%)	2 (5.4)	52 (20)	0.0179
Atrial fibrillation, n (%)	20 (54)	109 (41)	0.1341
Valvular heart disease, n (%)	22 (59)	110 (41)	0.0366
Cardiomyopathy, n (%)	3 (8)	43 (16)	0.1730
Hypertension, n (%)	25 (67)	181 (68)	0.9783
Diabetes mellitus, n (%)	11 (30)	97 (36)	0.4263
Dyslipidemia, n (%)	12 (32)	92 (34)	0.8071
Chronic kidney disease, n (%)	29 (81)	199 (75)	0.4862
History of heart failure, n (%)	13 (35)	102 (38)	0.7173
BMI, median [IQR]	23.3 [20.6, 25.3]	22.9 [20.4, 25.8]	0.8672
Systolic BP (mmHg), median [IQR]	156 [132, 173]	149 [123, 171]	0.2319
LVEF (%), median [IQR]	60 [58, 63]	35 [28, 45]	<0.0001
IVS (mm), median [IQR]	10 [9, 11.6 ]	10 [8.3, 11]	0.4114
IVC (mm), median [IQR]	17.5 [3.8, 20]	16 [10, 19.6]	0.6472
E/E′, median [IQR]	13.9 [10.7, 27.2]	14.8 [11.8, 20.8]	0.8055
Hemoglobin (g/dL), median [IQR]	10.8 [9.1, 12.4]	11.6 [9.9, 13.3]	0.0479
Albumin (g/dL), median [IQR]	3.3 [3.0, 3.6]	3.2 [2.9, 3.5]	0.2444
NT-proBNP (pg/mL), median [IQR]	2339 [1174, 5621]	8011 [3607, 1585]	<0.0001
ACEI/ARB, n (%)	16 (43)	86 (32)	0.1902
Beta-blocker, n (%)	12 (32)	93 (35)	0.7727
Diuretic, n (%)	23 (62)	111 (41)	0.0184
Low SMI, n (%)	27 (73)	130 (49)	0.0048
Edema index, median [IQR]	0.418 [0.399, 0.419]	0.402 [0.39, 0.416]	0.1635
NYHA 1/2/3/4, n (%)	0/0/12/9 (0/0/57/43)	0/2/90/89 (0/1/49/49)	0.6713

ACEI, angiotensin-converting enzyme inhibitor; ARB, angiotensin receptor blocker; BMI, body mass index; BP, blood pressure; HFnEF, heart failure with normal ejection fraction; IQR, interquartile range; IVC, inferior vena cava diameter; IVS, interventricular septum diameter; LVEF, left ventricular ejection fraction; NT-proBNP, N-terminal pro brain natriuretic peptide; NYHA, New York Heart Association functional class; SMI, skeletal mass index

**Table 2:** Results of univariate and multivariate analyses of factors associated with HFnEF

	Univariate analysis OR (95% CI)	p-value	Multivariate analysis OR (95% CI)	p-value
Age	1.05 (1.01–1.10)	0.0026	1.06 (1.02–1.11)	0.0010
Male sex	0.63 (0.31–1.26)	0.1930	0.86 (0.38–1.94)	0.7191
Coronary artery disease	0.24 (0.05–1.01)	0.0179	0.27 (0.05–1.31)	0.0676
Atrial fibrillation	1.69 (0.85–3.38)	0.1341		
Valvular heart disease	2.09 (1.04–4.22)	0.0366	2.00 (0.87–4.60)	0.1008
Cardiomyopathy	0.46 (0.14–1.56)	0.1730		
Hypertension	0.99 (0.47–2.06)	0.9783		
Diabetes mellitus	0.74 (0.35–1.56)	0.4263		
Dyslipidemia	0.91 (0.44–1.90)	0.8071		
Chronic kidney disease	1.35 (0.57–3.23)	0.4862		
History of heart failure	0.32 (0.06–1.73)	0.1587		
BMI	0.97 (0.90–1.04)	0.3940		
Systolic BP	1.01 (1.00–1.01)	0.2213		
IVS	1.13 (0.93–1.37)	0.2320		
IVC	1.02 (0.96–1.10)	0.4629		
E/E′	0.97 (0.90–1.04)	0.2872		
Hemoglobin	0.87 (0.76–1.01)	0.0688		
Albumin	1.64 (0.81–3.28)	0.1558		
NT-proBNP	1.00 (1.00–1.00)	<0.0001	1.00 (1.00–1.00)	<0.0001
ACEI/ARB	1.60 (0.80–3.22)	0.1902		
Beta-blocker	0.90 (0.43–1.87)	0.7737		
Diuretic	2.31 (1.14–4.68)	0.0184	2.32 (1.01–5.35)	0.0447
Low SMI	2.85 (1.32–6.11)	0.0048	2.92 (1.22–7.00)	0.0124
Edema index	3.90 (0.64–23.63)	0.1349		

Models adjusted for age, sex, coronary artery disease, valvular heart disease, diuretic therapy, NT-proBNP, and a low SMI score. ACEI, angiotensin-converting enzyme inhibitor; ARB, angiotensin receptor blocker; BMI, body mass index; BP, blood pressure; CI, confidence interval; IVC, inferior vena cava diameter; IVS, interventricular septum diameter; NT-proBNP, N-terminal pro brain natriuretic peptide; OR, odds ratio; SMI, skeletal mass index
